# Sibling relationships as a resource for coping with traumatic events

**DOI:** 10.1186/2193-1801-3-525

**Published:** 2014-09-15

**Authors:** Giovanna Perricone, Valentina Fontana, Sofia Burgio, Concetta Polizzi

**Affiliations:** Department of Psychological, Educational and Training Sciences, University of Palermo, Italy, Viale delle Scienze – Ed. 15, Palermo, 90128 Italy

**Keywords:** Sibling relationship, Trauma, Developmental resource, Developmental risk

## Abstract

The study investigated the correlation between the perception of sibling relationship to cope an adverse occurrence – the partial collapse of a primary school – and the indicators related to the traumatic impact set off by the event, by soliciting the child’s reminiscence of the catastrophic experience.

One hundred trauma-exposed children were recruited from a Sicilian primary school and were administered the following research instruments: the Trauma Symptom Checklist for Children (TSCC-A), to investigate the traumatized response that can be triggered in the children involved; the Brother as a Resource Questionnaire (BRQ), to delve into the perception of sibling relationship as a resource.

The outcomes showed statistically significant negative correlations between the Anxiety scale of the TSCC-A and the Scaffolding factors (r = −.260, p < .05) and Decision making process (r = −.315, p < 05) of the BRQ; between the Depression scale and the Scaffolding factors (r = −.147, p < .05), Emotional sharing (r = −.168, p < .05) and Decision making process (r = −.281, p < .05). The Anger scale correlated negatively with the Emotional sharing (r = −187, p < .05), the Decision making process (r = −.182, p < .05) and the Scaffolding factors (r = −.279, p < .05); the Post-traumatic Stress correlated negatively with the Scaffolding factors (r = −.203, p < .05) and the Decision making process (r = −.238, p < .05). Lastly, the Dissociation correlated negatively with the Decision making process (r = −.270, p < .05).

## Background

The study analysed sibling relationship as a resource for traumatized children. The adverse occurrence treated in this paper is the unexpected partial collapse of a school with an avoidable urged evacuation. The reaction set off by this accident may be considered tantamount to that experienced after a sudden natural catastrophe such as an earthquake or a flood.

This research article, which is in line with the extensive body of international and national literature dealing with this subject, wanted to shine a light on the sibling relationship regarded as a significant resource for the child’s and adolescent’s development process at cognitive, social and emotional levels (Dunn 1983; Dunn and Munn 1986; Dunn and Plomin 1990; Volling et al. 2004; Howe and Rinaldi 2004; Klein et al. 2003; Scabini and Infrate 2003; Teti 2001; Howe et al. 2011). Beyond being based on genetic inheritance and socio-family upbringing, the sibling relationship is built step by step by sharing experiences and reminiscences (Howe et al. 2001; Deater-Deckard et al. 2002). The literature of the field asserts the sibling bond plays a relevant role in shaping the child’s self-identity, as well as in developing the several cognitive, social and emotional capabilities such as the *perspective taking* process; the social comprehension; the management of social conflict; the negotiation among peers; the discover of the interpersonal relational styles – usable in their adulthood, too – and the ability of thinking about the events occurred. Such an important relationship also contributes to develop the *mind theory*; the empathic and sharing-feeling capability; the role taking process, etc. (Sulloway 1998; Volling and Blandon 2003; Dunn 2012; Lecce et al. 2002; Downey and Condron 2004; De Plano and Smorti 2007). At the level of social development, the *mirroring brain* process allows the child to manage the peer-to-peer relationship that will be a model for his future horizontal relationships (Sommantico and Tavazza 2006). Sibling companionship is also regarded as a form of alliance useful for better understanding and assigning a value to the events occurred (Scalisi 1995), and as a reciprocal supportive experience during difficult moments experienced by children (Howe et al. 2001). Moreover, some developmental factors, such as the child’s perception of the presence of his sibling during his mother’s pregnancy, account for the fact that it is one of the most significant relationships of a person’s life (Rufo 2004).

This paper analysed this relationship as a resource for the *scaffolding process* – a support to manage the elaboration, as well as to face the tasks related to the critical event – and for the emotional and supportive sharing in the *decision making* process.

On the basis of the theoretical reference model adopted, the survey considered the valence of the sibling relationship in relation to three functioning areas: a) the learning and development of cognitive skills such as the perception of the increase of the *perspective* and *role taking* capabilities – as the reciprocal ability of putting oneself in other people’s shoes (De Plano and Smorti 2007); b) the promotion of *coping* and *problem solving* strategies; c) the learning and development of socio-relational skills in terms of social comprehension, empathy and self-efficacy perceived (Dunn 2012). The relationship between siblings might be a source of social and emotional support useful for managing, understanding and sharing emotions.

The sufferance and fear provoked by a critical event make the recollecting process arduous to accomplish (Bahrick et al. 1998; Camisasca and Pirovano 2001; Di Blasio 2001). Moreover, this process may be either facilitated or inhibited in children by factors such as age, the effectiveness of the child’s role-model adult, the child’s capability to discern the different aspects of specific situations, the feeling experienced, and the child’s own personality (Camisasca and Pirovano 2001; Fivush 2001). Recollecting a trauma is a challenging action when the child cannot access to the memory of the event as freely as in case he wanted to reminisce happy moments.

Therefore, the question could be asked: after becoming victim of an unexpected sorrowful and/or fearful event, how considerable may be the role of a model figure like a sibling for a traumatized child? Sharing the reminiscences related to the adverse occurrence with his sibling, what perception has he had of their relationship? These questions stimulated and framed the research project.

## Method

### Objectives and hypothesis of the research

The study was aimed at investigating whether and how the sibling relationship has been perceived by the children involved in the trauma of an unexpected partial collapse of their school. It wants to verify the following hypothesis:

- whether the description of sibling relationship – reported by the child recollecting the experience of the traumatic event – met the behavioural pattern model that conceives it as a resource to cope a trauma.
- whether the description of the sibling relationship as a resource to cope the traumatic event showed statistically significant gender-differences.
- whether there were statistically significant correlations between the factors detecting the sibling relationship as a resource and the indicators related to the traumatic impact of the catastrophic event.
- whether the reaction set off by the catastrophic event showed statistically significant gender-differences.

### Participants

It was recruited a group of one hundred children who had been involved in the traumatic event of the partial collapse of their school, and consequently transferred to another school. All of them were between the ages of 9 to 11 (average age = 9.98; standard deviation [SD] = .696), and they were attending the fourth and fifth year at the regional primary school. 46% were males while 54% females, 61% of which were firstborn (Table 1).

**Table 1:** **The characteristics of the group of children (N = 100)**

Variable	Mean	SD
**Age**	9.98	.696
**Birth order:**		
*Firstborn %*	61%	
*Younger %*	30%	
*Twin %*	9%	
**Gender:**		
*Females %*	54%	
*Males %*	46%	

The study was carry out in compliance with the Helsinki Declaration. Moreover, the study was not accomplished in accordance with the ethics committee approval, considering that the research was carry out into some educational paths provided by the school during the academic year. In particular, the study was carry out after the convocation of the school board and the approval by the teachers and the principal of the school. The study was performed after having obtained the written informed consent declaration by the parents of the children involved.

### Measures and procedures

The children were solicited to reminisce the event experienced, namely the sudden partial collapse of their school, through a work discussion where they had to report the time, the place, the actions, and the subjects involved, focalizing on their personal emotions and, at the same time, presuming the others. This practice was the starting-point for reflections on the death risk implied by the event, and on the possible impairing organic and/or psychological response to trauma, such as experiencing a continuous sense of anxiety in classroom, or being often on the alert during the day. Most children, in effect, are usually unable to manage on their own the anxiety that would emerge when they reminisce the event experienced.

The following specific tools of detection were administered:

- ●The *Trauma Symptom Checklist for Children (TSCC-A)* (Briere, 2011), to evaluate the likely posttraumatic symptomatology of the children involved in the partial collapse of their school. The checklist is aimed at children/adolescents between the ages of 8 to 16 – for the detection of a cluster of psychological consequences that might have been triggered by traumatic events such as physical and sexual abuse, major loss, peer-to-peer bullying, and experiencing the ravages of natural disaster. It is a self-report 44-item instrument with Likert-scale 4-point responses ranging from 0 to 3, and yields two validity scales (Underresponse and Hyperresponse) and five clinical scales (Anxiety, Depression, Anger, Post-traumatic Stress, and Dissociation). The Anxiety scale includes items that measure the grade of the child’s generalized anxiety, hyperactivity, worries and fear. High scores would reveal either anxiety or hyperactivity problems related to the post-traumatic stress disorder. The Depression scale consists of items pertaining feelings of sadness, unhappiness, loneliness, etc., while the Anger scale is made up of items detecting feelings, thoughts and behaviour expressing anger; high scores would show the presence of aggressive and hostile behaviours. The items of the Post-traumatic scale are referred to specific post-traumatic symptoms such as intrusive thoughts, sensations and memories of early sorrowful events that can bring children either anxious distraction or irritability. The Dissociation scale, lastly, includes items that measure the possible dissociative symptomatology; high scores would display diminished sensitiveness toward the environment, emotional detachment and the tendency to remove any affective aspects at cognitive level. The instrument exhibits a good level of validity (range from .55 to .88) as for psychometric propriety.
- ●*The Brother as a Resource Questionnaire (BRQ),* is an instrument designed and validated by the Research Unit of Paediatric Psychology of the Psychological, Pedagogical and Educational Sciences Department of the University of the Studies of Palermo. It is aimed at investigating the individual child perception of the sibling relationship as a resource, by soliciting the reminiscence of the traumatic event experienced. It is a self-report 21-item questionnaire that implies 3-point responses of the Likert scale (1 = never; 2 = sometimes; 3 = always). The major factors investigated were:

- *Sustaining the accomplishment of a task (scaffolding)*: it measures how the sibling promotes the development of specific competences, and how he helps the traumatized youngster when he has to face a critic event.
- *Emotional sharing*: it determines the way a sibling may be a support when sorrowful and difficult moments are experienced.
- *Decision making during recreational activities*: it assesses how useful resource a sibling may be when the child has to take a decision related mainly to recreational activities engaging siblings.

The psychometric characteristics of the instrument such as reliability and validity were assessed by different methods. The value of Cronbach’s alpha – the coefficient of internal consistency – was 0.873. The applicability of the factorial analysis was assessed by dint of both Kaiser-Meyer-Olkin test (KMO) that provided an index value of 0.877, and Bartlett’s test of Sphericity whose index was null.

### Data treatment and analysis

The data obtained were codified according to the reference procedures of each instrument that was surveyed firstly using the descriptive analysis and, subsequently, the Statistical Package for the Social Sciences (SPSS) (Version19 for Windows) for the parametrical statistics. Pearson’ s coefficient of correlation was used to verify the existence of possible statistically significant correlations between each factors of the sibling relationship as a resource (i.e. Scaffolding, Emotional sharing, Decision making) and the indicators of the traumatic condition related to the catastrophic event (i.e. Anxiety, Depression, Anger, Post-traumatic Stress, Dissociation). An analysis of the variance (ANOVA) was also performed to detect any possible statistically significant gender differences in relation to the perception of the sibling relationship as a resource, as well as to the traumatic impact of the event experienced.

## Results

61% of the children involved in the study indicated his elder sibling as a useful resource to face the catastrophic event experienced. On the basis of such data, it can be inferred that the elder brother would play a considerable supporting role in sharing the most difficult moments of a child’s life.

The scores obtained by the TSCC-A scales, which measured the traumatic impact of the catastrophic event experienced, and those relating to BRQ factors, which described the perception of the sibling relationship as an effective resource to cope the trauma, showed several interesting statistically significant negative correlations. These are:

among the Anxiety scale of the TSCC-A, the Scaffolding factors (r = −.260^**^) and Decision making (r = −.315^**^) of the BRQ, as well as among the Depression scale and the factors of the Scaffolding (r = −.147), Emotional sharing (r = −.168) and Decision making (r = −.281^**^); while the Anger scale correlates with Emotional sharing (−187), Decision making (r = −.182) and Scaffolding (r = −.279^**^); the Post-traumatic Stress always correlates negatively with Scaffolding (r = −.203^**^) as well as with Decision making (r = −.238^**^); Dissociation correlates with Decision making (r = −.270^**^) (Table 2).

**Table 2:** **Correlations between the factors of the sibling relationship and the indicators related to the factors of the traumatic event**

Variables	Anxiety	Anger	Depression	Post-traumatic stress	Dissociation
**Scaffolding**	-.260**	-.279**	-.147	-.203**	
**Emotional sharing**	-.315**	-.187	-.168		
**Decision making**		-.182	-.281**	-.238**	-.270**

**p < .05.

In relation to the descriptive analysis, the results gained through the TSCC-A showed that the catastrophic event experienced by the children triggered a post-traumatic reaction displayed by recurrent intrusive and sorrowful thoughts (M = 8.8), signs of depression such as deep sense of sadness, loneliness, etc. (M = 6.16) and generalized anxiety (M = 5.91) (Table 3).

**Table 3:** **Descriptive statistics**

***Variables***	***Mean***	***SD***
**TSCC-A**		
Anxiety	5.91	4.22
Anger	4.7	4.26
Depression	6.16	3.96
Post-traumatic stress	8.8	5
Dissociation	5.51	4.31
**BRQ**		
Scaffolding	20.86	5.19
Emotional sharing	6.31	1.74
Decision making	4	1.49

The descriptive data regarding the perception of the sibling relationship as a resource stressed the relevance of the Scaffolding factor (M = 20.86) (Table 3).

The outcomes of the analysis of the possible gender differences, performed via the TSCC-A clinical scales, indicated that males only scored highly than females in the Anger scale (F = 9.48; p < .05) (Table 4), albeit on average they both scored higher than the reference normative population. The same standard deviation was also found in the results of the Depression scale.

**Table 4:** **Analysis of the variance (ANOVA) of the gender differences in relation to the clinical scales (TSCC-A) and the perception of the sibling relationship (BRQ)**

	Males		Females			
Variables	***Mean***	***Ds***	***Mean***	***Ds***	***F***	***Sig***
**(TSCC-A)**						
Anxiety	5.76	4.02	6.04	4.41	.105	.746
Depression	6.54	4.07	5.83	3.87	.797	.374
Anger	6.07	4.79	3.54	3.38	9.48	**.003**
Post-traumatic stress	8.76	4.93	8.83	5.12	.005	.943
Dissociation	6.20	4.44	4.93	4.15	2.17	.143
**(BRQ)**						
Scaffolding	20.13	4.86	21.48	5.43	1.69	.196
Emotional sharing	6.28	1.94	6.33	1.58	.021	.886
Decision making	3.93	1.51	4.22	1.47	.922	.339

On the contrary, no significantly gender difference was found with regard to the perception of the sibling relationship as resource (Table 4).

## Conclusions

The aspects of the issue stemmed from the results have given birth interesting considerations that, besides being in line with the literature of the field, have provided a series of relevant elements useful to put forward innovative heuristic hypothesis.

The first interesting outcome, even though only descriptive, is the evident valence of the sibling relationship – mostly with elder brothers – as an effective resource, especially in terms of scaffolding. It emerges how considerable is the role of a brother – especially the elder – as a guidance for his sibling in coping traumatic events. Effectively, facing the reaction set off by a traumatic event, and realizing what really happened, requires a very challenging effort at emotional, cognitive and practical levels as well.

It seems to be also newsworthy the result related to the gender-different reaction to the event analysed, which ascribed higher levels of anger and stifled emotional stress in males (Karos et al. 2007).

As for the statistically significant negative correlations, those between the Anxiety scale and the Scaffolding and Decision-making factors lead to hypothesize that, the more considerable is the perception of helpfulness of the sibling relationship as a resource, the lower is the anxiety level suffered by the child who experienced the event. Hence, both support and decision-making process could play a mediational role for the anxiety factor.

The results relating to the correlations between the scales of Depression and Anger, and the three factors of the sibling relationship as a resource (i.e. Scaffolding, Emotional Sharing, and Decision making), seem to show that the complexity in itself of the relationship – founded on cognitive and affective factors – would promote a restraint of emotional feelings (Howe et al. 2001).

The data regarding the correlation between the Post Traumatic Stress scale and the Scaffolding and Decision Making factors, as well as that between the Dissociation scale and Decision Making factor, suggest that the possibility for a child to be guided by a sibling in the elaboration of tasks, in decision-making and problem solving processes, seem to be very helpful to cope the post traumatic stress and its consequent risk of not being able to assimilate the experience into his own history. Hence, the sibling relationship could be the keystone to go beyond the ineluctability of the event itself, not excluding and/or avoiding it, but redefining it as a critic moment in his life, rather than as impairment. Moreover, such considerations would seem to be in line with some recent studies on the developmental potentialities of the sibling relationship (Lecce et al. 2007; Lecce and Pinto 2004). The final point to stress is the correlation between the factors of the sibling relationship of the BRQ and the levels of the scores of the TSCC-A scale, whereas the high scores obtained by the BRQ would suggest that the perception of the sibling relationship induce some reference models that allow the processes investigated through the clinical scales to be kept at low levels.

To recapitulate, it seems that the sibling relationship is a real resource to be protected especially during difficult moments. In particular, it can be thought that the children involved, while reminiscing the quality of the bond with their own brother in respect of the event experienced, had gave it great credit and, therefore, they had perceived the elder brother in terms of scaffolding, emotional support and as reference model from whom they have learned new ways of coping the critical event. The sibling subsystem, effectively, “is the first social laboratory where children learn to negotiate, cooperate and compete with each other” (Minuchin 1976).

This research paper, even though in line with the reference national and international literature of the field, seems to provide innovative aspects regarding further studies, as well as intervention/treatment guides in the field of the Psychology of Emergence.

The first development plan – undertaken by the authors – could be the possibility to use the frame of the research project to investigate different conditions of trauma that imply the involvement of family system such as conditions of trauma-illness, trauma related to invasive and/or non-invasive pharmacology treatment, that often give birth to real developmental post-traumatic disorder (Perricone Briulotta 2012; Perricone et al. 2012; Perricone et al. 2013a, [b]; Perricone et al. 2010a, [b]). Moreover, the study allows to hypothesize intervention of psychological rehabilitation and specific trainings including methodological field research, that deals with the sibling relationship as a resource.

### Consent

Children were involved after their parents had signed the declaration of informed consent according to the D.LGS. 196/2003 art.13 related to their personal and other people’s data protection. Written informed consent was obtained from parents for publication of this report.
